# The association of pancreatic cancer incidence with smoking status and smoking amount in Korean men

**DOI:** 10.4178/epih.e2022040

**Published:** 2022-04-21

**Authors:** Do Jin Nam, Chang-Mo Oh, Eunhee Ha, Min-Ho Kim, Eun Hye Yang, Hyo Choon Lee, Soon Su Shin, Woo Yeon Hwang, Ann Hee You, Jae-Hong Ryoo

**Affiliations:** 1 Department of Occupational and Environmental Medicine, Kyung Hee University Medical Center, Seoul, Korea; 2 Department of Preventive Medicine, Kyung Hee University School of Medicine, Seoul, Korea; 3 Department of Occupational and Environmental Medicine, Ewha Womans University College of Medicine, Seoul, Korea; 4 Department of Obstetrics and Gynecology, Kyung Hee University Hospital, Seoul, Korea; 5 Department of Anesthesiology and Pain Medicine, Kyung Hee University Hospital, Seoul, Korea; 6 Department of Occupational and Environmental Medicine, Kyung Hee University School of Medicine, Seoul, Korea

**Keywords:** Smoking, Pancreatic cancer, Smoking cessation

## Abstract

**OBJECTIVES:**

Our study examined the dose-response relationship between smoking amounts (pack-years) and the risk of developing pancreatic cancer in Korean men.

**METHODS:**

Of 125,743 participants who underwent medical health checkups in 2009, 121,408 were included in the final analysis and observed for the development of pancreatic cancer. We evaluated the associations between smoking amounts and incident pancreatic cancer in 4 groups classified by pack-year amounts. Cox proportional hazards models were used to estimate the adjusted hazard ratios (HRs) and 95% confidence intervals (CIs) of incident pancreatic cancer by comparing groups 2 (<20 pack-year smokers), 3 (20-≤40 pack-year smokers), and 4 (>40 pack-year smokers) with group 1 (never smokers).

**RESULTS:**

During 527,974.5 person-years of follow-up, 245 incident cases of pancreatic cancer developed between 2009 and 2013. The multivariate-adjusted HRs (95% CIs) for incident pancreatic cancer in groups 2, 3, and 4 were 1.05 (0.76 to 1.45), 1.28 (0.91 to 1.80), and 1.57 (1.00 to 2.46), respectively (p for trend=0.025). The HR (95% CI) of former smokers showed a dose-response relationship in the unadjusted model, but did not show a statistically significant association in the multivariate-adjusted model. The HR (95% CI) of current smokers showed a dose-response relationship in both the unadjusted (p for trend=0.020) and multivariate-adjusted models (p for trend=0.050).

**CONCLUSIONS:**

The risk of developing pancreatic cancer was higher in current smokers status than in former smokers among Korean men, indicating that smoking cessation may have a protective effect.

## INTRODUCTION

Smoking is a widely practiced habit, and smoking rates vary by country and gender [1]. In 2019, the smoking rates in the United States and the United Kingdom were 10.4% and 15.8%, respectively [2]. Among the Korean population in 2019, 35.7% of men and 6.7% of women were smokers [3]. Pancreatic cancer is the 11th most common type of cancer in the world, with 458,918 cases and 432,242 deaths recorded in 2018, accounting for approximately 4.5% of deaths due to cancer [1]. It is the eighth most common type of cancer in Korea and the fifth most common cause of death due to cancer, with 7,032 deaths (3,733 men and 3,299 women) reported in 2017 and 6,036 deaths (3,193 men and 2,843 women) in 2018 [4]. The major factors associated with pancreatic cancer include smoking and a family history of pancreatic cancer [5]. It is presumed that smoking causes pancreatic cancer through the transportation of nitrosamine (a carcinogen in cigarettes) to the pancreas by blood or bile [6]. Since the 5-year survival rate for pancreatic cancer is very low (12.2%; 11.8% in men and 12.5% in women) [7], it is crucial to manage and prevent smoking. Many studies have been conducted on the risk of pancreatic cancer based on smoking status, including research targeting much of the European population [8,9] and another study conducted in hospitals in northern Italy [10]. However, research on this topic has not been actively conducted in Asia. We also found few studies on the relationship of smoking cessation to changes in the risk of developing pancreatic cancer. Therefore, this study evaluated the risk of developing pancreatic cancer according to the amount smoked and smoking status (current or former) in Korean men.

## MATERIALS AND METHODS

### Data sources

The national health insurance system of Korea covers 97% of the population. Therefore, its database is representative of the medical service usage of the overall Korean population [11]. Additionally, most Koreans over age 40 years are required to undergo a medical health checkup at least once every 2 years. Data from these medical health checkups are collected and stored by the National Health Insurance Corporation (NHIC) in Korea. These data were used to establish a cohort study of approximately 510,000 health insurance participants from 2002-2015 (14 years) and were categorized according to participants’ characteristics and income information (social and economic variables), health examination results, and nursing institution information. This cohort study included 10% of adults between 40 years and 79 years who received general health examinations in 2002 [12] and followed them for an additional 13 years. Diagnoses were coded according to the International Classification of Diseases, 10th revision (ICD-10) [13]. The NHIC in Korea recently began providing the sampled database for research purposes after deleting personal identification information. This sampled database includes information from health checkups linked to pancreatic cancer diagnoses as recorded in Statistics Korea.

### Study participants

**Figure 1.**
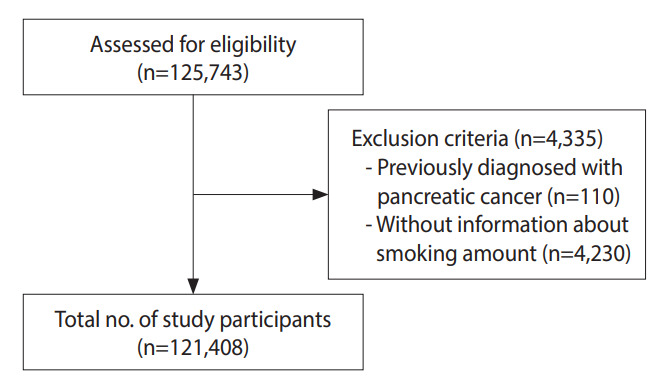
Flow chart of enrolled study participants.

In total, 125,743 men who underwent health checkups in 2009, according to the National Health Information database, were included in the present analysis. We excluded 110 individuals who had been diagnosed with pancreatic cancer (ICD-C25) between 2002 and the health examination in 2009. An additional 4,230 individuals without information on smoking amounts were also excluded. Considering some overlap in exclusion criteria, 121,408 men were included in the final analysis and observed for the development of pancreatic cancer (Figure 1). Pancreatic cancer as a primary diagnosis was the criterion used to include newly diagnosed cases.

### Health survey examinations and laboratory measurements

The health examinations included a questionnaire on lifestyle and past medical history. Participants were categorized by smoking status as never smokers, former smokers, and current smokers. The smoking amount was expressed in pack-years, which was calculated using the equation: 1 pack-year=1 year× 1 pack per day. The participants were divided into 4 groups based on the smoking amount: group 1, never smokers; group 2, 0-19 pack-year smokers; group 3, 20-40 pack-year smokers; and group 4, > 40 packyear smokers. Alcohol intake was defined as intake > 3 times per week. Physical activity was defined as performing moderate-intensity physical activity at least 30 minutes per day for more than 4 days each week or vigorous-intensity physical activity at least 20 minutes per day for more than 4 days each week [14]. Body mass index (BMI) was calculated as weight (kg) divided by height squared (m^2^). Systolic blood pressure (BP) and diastolic BP were measured by trained examiners. Laboratory data collected from the health examinations included fasting blood glucose, total cholesterol, triglyceride, high-density lipoprotein (HDL) cholesterol, low-density lipoprotein (LDL) cholesterol, and serum creatinine (SCr). Kidney function was assessed by the estimated glomerular filtration rate (eGFR) calculated using the Chronic Kidney Disease Epidemiology Collaboration equation as follows:

eGFR=141 × min (SCr/*K*, 1)^a^ × max (SCr/*K*, 1)^-1.209^ × 0.993^age^ × 1.018 (if women) × 1.159 (if Black)

where, “*K*” is 0.7 for women and 0.9 for men, “a” is -0.329 for women and -0.411 for men, “min” is the minimum SCr/*K* or 1, and “max” is the maximum SCr/*K* or 1 [15].

### Outcome definitions

This study began on the date of the first health checkup in 2009, and the last follow-up date for the diagnosis of pancreatic cancer was December 31, 2013. The incidence of pancreatic cancer was determined by identifying participants who were newly diagnosed with pancreatic cancer during the follow-up period, based on the ICD-10 code C25 (C25.0-25.9) as registered in the NHIC and linked to the disease diagnosis data in Statistics Korea. The primary clinical endpoint of interest was the development of pancreatic cancer, which was included in the composite endpoint of the study. The total follow-up period was 527,974.5 person-years, and the average follow-up period was 4.35 (standard deviation [SD], 0.48) person-years.

### Statistical analysis

Data were expressed as mean±SD or medians (interquartile ranges) for continuous variables and as numbers and percentages for categorical variables.

One-way analysis of variance and the chi-square test were used to analyze statistical differences among the characteristics of the participants according to the 4 pack-year classification groups at the time of enrollment.

Sensitivity analysis was performed after adjusting the cut-off value for smoking amount and re-classifying the 4 groups.

Person-years were calculated as the sum of the follow-up times from the baseline until the time of a pancreatic cancer diagnosis or until December 31, 2013.

To evaluate the association between smoking amount and incident pancreatic cancer, we used Cox proportional hazards models to estimate the adjusted hazard ratios (HRs) and 95% confidence intervals (CIs) for incident pancreatic cancer by comparing groups 2, 3, and 4 with group 1. The Cox proportional hazard models were adjusted for multiple confounding factors. In the multivariate models, we included variables that might confound the relationship between the smoking amount and incident pancreatic cancer, such as age, BMI, systolic BP, fasting blood glucose, total cholesterol, eGFR, alcohol intake, and physical activity. To test the validity of the Cox proportional hazard models, we checked the proportional hazard assumption, which was assessed by the logminus-log survival function and graphically found to be unviolated. A p-value < 0.05 was considered statistically significant. All statistical analyses were performed using SAS version 9.4 (SAS Institute Inc., Cary, NC, USA).

### Ethics statement

Ethics approval for the study protocol and data analysis was obtained from the Institutional Review Board of Kyung Hee University Hospital (IRB No. 2018-12-020). Informed consent was waived because a retrospectively anonymized database was used for analysis.

## RESULTS

During the 527,974.5 person-years of follow-up, 245 (0.20%) incident cases of pancreatic cancer developed between 2009 and 2013. The baseline characteristics of the participants according to the 4 groups are presented in Table 1. At baseline, the mean±SD age and BMI of participants were 57.5±8.6 years and 24.0±2.8 kg/m^2^, respectively. There were significant differences among all the listed variables in the 4 groups, except for systolic BP.

Table 2 shows the HRs and 95% CIs for incident pancreatic cancer according to the 4 groups. In the unadjusted model, the HRs and 95% CIs for incident pancreatic cancer when comparing groups 2, 3, and 4 with group 1 were 0.76 (0.56 to 1.03), 1.05 (0.75 to 1.46), and 1.84 (1.18 to 2.86), respectively (p for trend=0.001).

These associations remained statistically significant, even after further adjustments for covariates in the multivariate-adjusted model, where the adjusted HRs and 95% CIs for incident pancreatic cancer in groups 2, 3, and 4 were 1.05 (0.76 to 1.45), 1.28 (0.91 to 1.80), and 1.57 (1.00 to 2.46), respectively (p for trend=0.025).

The smoking status subgroup analysis indicated that current smoking was significantly associated with an increased risk of incident pancreatic cancer even after adjusting the covariates (p for trend=0.050). Former smoking also showed a significant association in the unadjusted model (p for trend=0.019) but not after adjustment for covariates (p for trend=0.152) (Table 3).

Similar results were confirmed after adjusting the cut-off values for the smoking amount (15 pack-years and 25 pack-years) (Supplementary Materials 1 and 2). Additional adjustment using the Charlson comorbidity index also found similar results (Supplementary Materials 3 and 4).

## DISCUSSION

In this study, the associations of pancreatic cancer risk with the amount of smoking and with cessation of smoking were retrospectively confirmed by comparing current smokers, former smokers, and never smokers. Compared to never smokers, the risk of developing pancreatic cancer increased in smokers as the amount of smoking increased. However, in former smokers, the risk of developing pancreatic cancer was not statistically significantly elevated, unlike what was observed for current smokers. Conversely, the risk of developing pancreatic cancer was found to be higher when current smokers had high pack-years. Smoking is known worldwide as a cause of pancreatic cancer, and studies comparing former smokers and current smokers are being actively conducted.

A study involving 465,910 people in Europe showed that longer durations of smoking and higher daily smoking amounts increased the subsequent risk of pancreatic cancer [9]. The same results were found in a study conducted on Lithuanian men [16], and a hospital-based case-control study conducted in Italy showed that early smoking cessation lowered the risk of pancreatic cancer [10]. A cohort study that established smoker categories based on blood cotinine levels from the 1986 to 2013 Health Professionals Follow-Up Study and the Nurses’ Health Study showed that higher smoking amounts (i.e., higher cotinine levels) were associated with higher mortality rates in pancreatic cancer patients [17]. Several of these findings were consistent with those of our study. Furthermore, our study also showed that the risk of developing pancreatic cancer could be lowered by smoking cessation.

There are 3 major biological mechanisms by which smoking causes the development of pancreatic cancer. First, cell differentiation and angiogenesis are induced by promoting the expression of K-ras and matrix metalloproteinase 7, a cell differentiation promoting factor, by causing genetic variation. Second, smoking activates the immune system, including neutrophils, large families, and T-cells, causing chronic inflammation and fibrosis and thereby leading to pancreatic cancer. Third, nicotine intake increases the secretion and production of digestive enzymes in the body, and pancreatic cancer can be caused by the activation of the secretory cells in the pancreas [18].

Pancreatic cancer is difficult to diagnose in the early stages and is most often diagnosed in the advanced stage, for which reason it has a high mortality rate [1]. Therefore, prevention is critical for pancreatic cancer. Smoking cessation policies at the national level have been actively implemented since the 2000s under Article 9 of the National Health Promotion Act, and the smoking rate in Korea has shown a gradual decline from 27.8% in 2008 to 21.5% in 2019. Notably, the smoking rate among men decreased by more than 10%p from 47.8% in 2008 to 35.7% in 2019 [3]. In addition to the general health examination program, Korea is currently trying to lower the smoking rate through programs such as smoking prevention in minors, smoking cessation clinics in public health centers, and smoking cessation camps. Legal sanctions such as the designation of no smoking areas and increases in cigarette prices have also been implemented. Reduction in smoking can eventually reduce the risk of developing pancreatic cancer, as well as the related social, economic, and medical losses.

This study had some limitations. First, the study comprised only men participants and did not derive results for women. Second, as a survey-based investigation, it did not identify other factors that could have affected the development of pancreatic cancer, such as a family history of pancreatic cancer. Third, since survey responses depended on the examinee’s memory, bias due to inaccurate memories of smoking duration, intensity, and cessation was possible. Fourth, the subtypes of pancreatic cancer were not distinguished. Fifth, the period of smoking cessation was not reflected in the variables. Sixth, the national health insurance data used in this study were originally collected for claims purposes, not for research purposes. Therefore, the diagnoses may have been inaccurate because there are cases where a diagnosis code is included only for the purposes of applying for insurance reimbursement . Since secondary diagnoses were excluded, there is a possibility that the incidence of pancreatic cancer in this study reflected a reduced rate. Seventh, bias from follow-up loss may have affected our results. Participants lost to follow-up and not included in the analysis (n=4,230) were significantly different in age; triglyceride, LDL-cholesterol, SCr, and eGFR values; and alcohol intake compared to the analytic cohort. (Supplementary Material 5) Some follow-up loss is to be expected, especially in those who are in poor health, and the follow-up loss of high-risk people can lead to a conservative bias and subsequent underestimation of risk.

Despite these limitations, this study identified the risk of pancreatic cancer according to smoking amounts and smoking status using representative data of Korean men. It also confirmed that current smokers can reduce their risk of pancreatic cancer if they quit smoking. As a major risk factor for pancreatic cancer, it is important to manage smoking by continuing to implement smoking cessation policies.

In conclusion, this study identified the relationships between the amount of smoking, smoking status, and the risk of pancreatic cancer in Korean men. A higher number of smoking exposure (pack-years) were associated with a higher risk of developing pancreatic cancer, and unlike former smokers, current smokers showed a statistically significant risk of developing pancreatic cancer. According to the results of this study, the smoking rate needs to be lowered even further by implementing strong smoking cessation policies. Additional research is necessary to further clarify the risk of developing pancreatic cancer based on the amount of smoking and smoking status.

## Figures and Tables

**Table 1.** Baseline characteristics of men participants according to 4 smoking amount groups (n=121,408)

Characteristics	Overall	Smoking amount (pack-year)				
		Group 1 (non-smoker, n=43,387)	Group 2 (>0, ≤20, n=45,472)	Group 3 (>20, ≤40, n=26,015)	Group 4 (>40, n=6,534)	p for trend^1^
Person-year (total)	527,974.5	189,101.4	197,706.1	112,904.4	28,262.6	
Person-year (average)	4.35±0.48	4.36±0.51	4.35±0.43	4.34±0.49	4.32±0.60	<0.001
Age (yr)	57.5±8.6	59.1±9.2	55.6±7.8	57.2±7.9	60.9±9.1	<0.001
BMI (kg/m^2^)	24.0±2.8	24.1±2.8	24.0±2.7	23.9±2.8	24.0±3.0	0.026
Systolic BP (mmHg)	126.4±14.7	127.0±15.0	126.0±14.3	125.9±14.7	127.0±15.2	0.524
Diastolic BP (mmHg)	78.8±9.8	79.0±9.9	78.9±9.7	78.5±9.7	78.5±9.9	<0.001
Total cholesterol (mg/dL)	195.7±36.5	193.5±35.9	196.8±36.3	197.3±37.3	195.9±38.6	<0.001
Triglyceride (mg/dL)	126 (88-184)	116 (82-169)	127 (88-186)	135 (94-197)	139 (98-204)	<0.001
HDL-cholesterol (mg/dL)	53.2±29.9	53.9±33.7	52.9±25.4	52.7±29.5	52.4±32.8	<0.001
LDL-cholesterol (mg/dL)	113.9±38.5	113.8±38.9	114.5±37.6	113.5±39.3	111.4±38.9	<0.001
Fasting blood glucose (mg/dL)	103.1±27.5	102.6±26.9	102.7±26.6	103.9±28.6	106.1±31.5	<0.001
SCr (mg/dL)	1.32±1.74	1.28±1.65	1.40±1.90	1.31±1.65	1.16±1.54	<0.001
eGFR (mL/min per 1.73 m^2^)	80.0±20.9	79.4±20.2	80.1±21.9	80.6±20.8	80.2±18.7	<0.001
Charlson comorbidity index	2.43±2.17	2.62±2.26	2.19±2.03	2.41±2.15	2.98±2.35	<0.001
Smoking amount (pack-year)	13.8±16.2	0.0	11.5±6.2	30.2±5.5	56.6±15.8	<0.001
Smoking status						<0.001
Never smoker	43,387 (35.7)	43,387 (100)	0.0	0.0	0.0	
Former smoker	39,515 (32.6)	0.0	27,252 (69.0)	9,622 (24.3)	2,641 (6.7)	
Current smoker	38,506 (31.7)	0.0	18,220 (47.3)	16,393 (42.6)	3,893 (10.1)	
Alcohol intake	23.6	15.4	24.1	32.8	37.7	<0.001
Physical activity	18.3	18.6	19.1	16.9	15.5	<0.001
Development of pancreatic cancer	245 (0.20)	91 (0.21)	72 (0.16)	57 (0.22)	25 (0.38)	0.001

Values are presented as mean±standard deviation, medians (interquartile range), number (%), or percentages. BMI, body mass index; BP, blood pressure; HDL, high density lipoprotein; LDL, low density lipoprotein; SCr, serum creatinine; eGFR, estimated glomerular filtration rate.

1 The p-value by ANOVA test for continuous variables and chi square test for categorical variables.

**Table 2.** HRs and 95% CIs for the incidence of pancreatic cancer according to 4 smoking amount groups

Variables		Person-year	Incidence cases	Incidence density (per 10,000 person-year)	HR (95% CI)	
					Unadjusted	Multivariate adjusted model^1^
Smoking amount (pack-year)						
	Group 1 (never smoker)	189,101.4	91	4.8	1.00 (reference)	1.00 (reference)
	Group 2 (>0, ≤20)	197,706.1	72	3.7	0.76 (0.56, 1.03)	1.05 (0.76, 1.45)
	Group 3 (>20, ≤40)	112,904.4	57	5.0	1.05 (0.75, 1.46)	1.28 (0.91, 1.80)
	Group 4 (>40)	28,262.6	25	8.8	1.84 (1.18, 2.86)	1.57 (1.00, 2.46)
p for trend					0.001	0.025
Age		-	-	-	-	1.08 (1.07, 1.20)
BMI		-	-	-	-	0.99 (0.95, 1.04)
Systolic blood pressure		-	-	-	-	1.00 (0.99, 1.01)
Fasting blood glucose		-	-	-	-	1.01 (1.00, 1.01)
Total cholesterol		-	-	-	-	1.00 (1.00, 1.00)
eGFR		-	-	-	-	1.00 (0.99, 1.00)
Alcohol intake		-	-	-	-	1.27 (0.95, 1.68)
Physical activity		-	-	-	-	0.69 (0.48, 0.99)

HR, hazard ratio; CI, confidence interval; BMI, body mass index; eGFR, estimated glomerular filtration rate.

1 Multivariate adjusted model was adjusted for age, BMI, systolic BP, fasting blood glucose, total cholesterol, eGFR, alcohol intake and physical activity.

**Table 3.** The incidence of pancreatic cancer according to 4 smoking amount groups and smoking status subgroups

Variables		Former-smoker+never-smoker group (n=82,902)		Current smoker+never smoker group (n=81,893)	
		Unadjusted	Multivariate adjusted model^1^	Unadjusted	Multivariate adjusted model^1^
Smoking amount (pack-year)					
	Group 1 (never smoker)	1.00 (reference)	1.00 (reference)	1.00 (reference)	1.00 (reference)
	Group 2 (>0, ≤20)	0.73 (0.51, 1.06)	0.99 (0.68, 1.44)	0.79 (0.52, 1.20)	1.16 (0.75, 1.80)
	Group 3 (>20, ≤40)	1.44 (0.95, 2.19)	1.52 (0.99. 2.32)	0.82 (0.54, 1.25)	1.10 (0.71, 1.72)
	Group 4 (>40)	1.82 (0.95, 3.50)	1.44 (0.74. 2.77)	1.85 (1.07, 3.20)	1.71 (1.00, 2.98)
p for trend		0.019	0.152	0.020	0.050
Age		-	1.08 (1.06, 1.10)	-	1.08 (1.06, 1.10)
BMI		-	0.98 (0.93, 1.04)	-	1.00 (0.95, 1.06)
Systolic BP		-	1.00 (0.99, 1.01)	-	1.00 (0.99, 1.01)
Fasting blood glucose		-	1.01 (1.01, 1.01)	-	1.00 (1.00, 1.01)
Total cholesterol		-	1.00 (0.99, 1.00)	-	1.00 (0.99, 1.00)
eGFR		-	1.00 (1.00. 1.00)	-	0.99 (0.98, 1.00)
Alcohol intake		-	1.12 (0.78, 1.62)	-	1.42 (1.00, 2.01)
Physical activity		-	0.61 (0.40, 0.95)	-	0.71 (0.45, 1.12)

Values are presented as hazard ratio (95% confidence interval). BMI, body mass index; BP, blood pressure; eGFR, estimated glomerular filtration rate.

1 Multivariate adjusted model was adjusted for age, BMI, systolic BP, fasting blood glucose, total cholesterol, eGFR, alcohol intake and physical activity.

## SUPPLEMENTARY MATERIALS

Supplementary Material 1. Hazard ratios (HRs) and 95% confidence intervals (CI) for the incidence of pancreatic cancer according to four groups of smoking amount levels in smoking status subgroups

Supplementary Material 2. Hazard ratios (HRs) and 95% confidence intervals (CI) for the incidence of pancreatic cancer according to four groups of smoking amount levels in smoking status subgroups

Supplementary Material 3. Hazard ratios (HRs) and 95% confidence intervals (CI) for the incidence of pancreatic cancer according to four groups of smoking amount levels

Supplementary Material 4. Hazard ratios (HRs) and 95% confidence intervals (CI) for the incidence of pancreatic cancer according to four groups of smoking amount levels in smoking status subgroups

Supplementary Material 5. Comparison between exclusion from analysis and inclusion in analysis
