# Supplementary material for: The association of pancreatic cancer incidence with smoking status and smoking amount in Korean men
Source: Epidemiol Health. 2022 Apr 21;44:e2022040. doi: 10.4178/epih.e2022040 (PMC9350416; doi:10.4178/epih.e2022040)
Supplement: Supplementary Material 1. — Hazard ratios (HRs) and 95% confidence intervals (CI) for the incidence of pancreatic cancer according to four groups of smoking amount levels in smoking status subgroups [file epih-44-e2022040-suppl1.docx]

**Supplementary Material 1. Hazard ratios (HRs) and 95% confidence intervals (CI) for the incidence of pancreatic cancer according to four groups of smoking amount levels in smoking status subgroups**

|  | HR (95% CI) | | | |
| --- | --- | --- | --- | --- |
|  | Former -smoker + never smoker group (n=82,902) | | Current smoker + never smoker group (n=81,893) | |
|  | Unadjusted | Multivariate adjusted model | Unadjusted | Multivariate adjusted model |
| **Smoking amount** |  |  |  |  |
| Group 1(Never smoker) | 1.00 (reference) | 1.00 (reference) | 1.00 (reference) | 1.00 (reference) |
| Group 2(>0, ≤15) | 0.673 (0.446-1.017) | 0.909 (0.594 -1.391) | 0.849 (0.533 -1.353) | 1.231 (0.756 -2.007) |
| Group3(>15, ≤40) | 1.251 (0.865-1.808) | 1.426 (0.981-2.073) | 0.782 (0.531 -1.150) | 1.081 (0.720 -1.623) |
| Group4(>40) | 1.821 (0.948-3.499) | 1.437 (0.745-2.774) | 1.855 (1.075-3.203) | 1.708 (0.987-2.982) |
| P for trend | 0.024 | 0.163 | 0.025 | 0.060 |

Multivariate adjusted model was adjusted for age, BMI, systolic BP, fasting blood glucose,total cholesterol, eGFR, alcohol intake and physical activity.
