# Supplementary material for: The association of pancreatic cancer incidence with smoking status and smoking amount in Korean men
Source: Epidemiol Health. 2022 Apr 21;44:e2022040. doi: 10.4178/epih.e2022040 (PMC9350416; doi:10.4178/epih.e2022040)
Supplement: Supplementary Material 2. — Hazard ratios (HRs) and 95% confidence intervals (CI) for the incidence of pancreatic cancer according to four groups of smoking amount levels in smoking status subgroups [file epih-44-e2022040-suppl2.docx]

**Supplementary Material 2. Hazard ratios (HRs) and 95% confidence intervals (CI) for the incidence of pancreatic cancer according to four groups of smoking amount levels in smoking status subgroups**

|  | HR (95% CI) | | | |
| --- | --- | --- | --- | --- |
|  | Former -smoker + never smoker group (n=82,902) | | Current smoker + never smoker group (n=81,893) | |
|  | Unadjusted | Multivariate adjusted model | Unadjusted | Multivariate adjusted model |
| **Smoking amount** |  |  |  |  |
| Group 1(Never smoker) | 1.00 (reference) | 1.00 (reference) | 1.00 (reference) | 1.00 (reference) |
| Group 2(>0, ≤25) | 0.839 (0.598-1.177) | 1.128 (0.795 -1.600) | 0.792 (0.540 -1.161) | 1.136 (0.759 -1.699) |
| Group3(>25, ≤40) | 1.271 (0.766-2.107) | 1.245 (0.748-2.072) | 0.832 (0.518 -1.337) | 1.127 (0.688 -1.846) |
| Group4(>40) | 1.821 (0.948-3.499) | 1.431 (0.741-2.762 | 1.855 (1.075-3.203) | 1.710 (1.000-2.989) |
| P for trend | 0.033 | 0.257 | 0.019 | 0.050 |

Multivariate adjusted model was adjusted for age, BMI, systolic BP, fasting blood glucose,total cholesterol, eGFR, alcohol intake and physical activity.
