# Supplementary material for: The association of pancreatic cancer incidence with smoking status and smoking amount in Korean men
Source: Epidemiol Health. 2022 Apr 21;44:e2022040. doi: 10.4178/epih.e2022040 (PMC9350416; doi:10.4178/epih.e2022040)
Supplement: Supplementary Material 3. — Hazard ratios (HRs) and 95% confidence intervals (CI) for the incidence of pancreatic cancer according to four groups of smoking amount levels [file epih-44-e2022040-suppl3.docx]

**Supplementary Material 3. Hazard ratios (HRs) and 95% confidence intervals (CI) for the incidence of pancreatic cancer according to four groups of smoking amount levels**

|  | Person-year | Incidence cases | Incidence density  (per 10,000 person-year) | HR (95% CI) ^*^ | |
| --- | --- | --- | --- | --- | --- |
|  |  |  |  | Unadjusted | Multivariate adjusted model |
| **Smoking amount** |  |  |  |  |  |
| Group 1(Never smoker) | 189,101.4 | 91 | 4.8 | 1.00 (reference) | 1.00 (reference) |
| Group 2(>0, ≤20) | 197,706.1 | 72 | 3.7 | 0.758 (0.556-1.032) | 1.059 (0.768-1.460) |
| Group 3(>20, ≤40) | 112,904.4 | 57 | 5.0 | 1.050(0.754-1.463) | 1.281(0.912-1.790) |
| Group 4(>40) | 28,262.6 | 25 | 8.8 | 1.840 (1.182-2.865) | 1.558 (0.995-2.440) |
| *P* for trend |  |  |  | 0.001 | 0.029 |
| Age |  |  |  |  | 1.076 (1.061-1.092) |
| BMI |  |  |  |  | 0.988 (0.944-1.035) |
| Systolic BP |  |  |  |  | 1.000 (0.992-1.009) |
| Fasting blood glucose |  |  |  |  | 1.007 (1.004-1.010) |
| Total cholesterol |  |  |  |  | 1.000 (0.996-1.003) |
| eGFR |  |  |  |  | 0.997 (0.990-1.003) |
| Charlson comorbidity index |  |  |  |  | 1.057 (1.002-1.115) |
| Alcohol intake |  |  |  |  | 1.283 (0.964-1.708) |
| Physical activity |  |  |  |  | 0.693 (0.484-0.992) |

Multivariate adjusted model was adjusted for age, BMI, systolic BP, fasting blood glucose,total cholesterol,eGFR, Charlson comorbidity index, alcohol intake and physical activity.
