# Supplementary material for: The association of pancreatic cancer incidence with smoking status and smoking amount in Korean men
Source: Epidemiol Health. 2022 Apr 21;44:e2022040. doi: 10.4178/epih.e2022040 (PMC9350416; doi:10.4178/epih.e2022040)
Supplement: Supplementary Material 4. — Hazard ratios (HRs) and 95% confidence intervals (CI) for the incidence of pancreatic cancer according to four groups of smoking amount levels in smoking status subgroups [file epih-44-e2022040-suppl4.docx]

**Supplementary Material 4. Hazard ratios (HRs) and 95% confidence intervals (CI) for the incidence of pancreatic cancer according to four groups of smoking amount levels in smoking status subgroups**

|  | HR (95% CI) | | | |
| --- | --- | --- | --- | --- |
|  | Former -smoker + never smoker group(n=82,902) | | Current smoker + never smoker group (n=81,893) | |
|  | Unadjusted | Multivariate adjusted model | Unadjusted | Multivariate adjusted model |
| **Smoking amount** |  |  |  |  |
| Group 1(Never smoker) | 1.00 (reference) | 1.00 (reference) | 1.00 (reference) | 1.00 (reference) |
| Group 2(>0, ≤20) | 0.734 (0.509-1.058) | 0.990 (0.679 -1.443) | 0.793(0.525 -1.198) | 1.196 (0.773 -1.849) |
| Group3(>20, ≤40) | 1.442 (0.950-2.190) | 1.502 (0.984-2.291) | 0.820(0.537 -1.253) | 1.127 (0.723 -1.754) |
| Group4(>40) | 1.821 (0.948-3.499) | 1.404 (0.727-2.711) | 1.855 (1.075-3.203) | 1.715 (0.990-2.999) |
| P for trend | 0.019 | 0.176 | 0.020 | 0.060 |
| Age |  | 1.074 (1.056-1.093) |  | 1.071 (1.052-1.090) |
| BMI |  | 0.981 (0.927-1.037) |  | 0.994 (0.940-1.051) |
| Systolic BP |  | 1.001 (0.991-1.011) |  | 1.001 (0.991-1.010) |
| Fasting blood glucose |  | 1.008 (1.006-1.011) |  | 1.003 (0.999-1.008) |
| Total cholesterol |  | 1.000 (0.995-1.004) |  | 0.999 (0.995-1.004) |
| eGFR |  | 0.998 (0.991-1.006) |  | 0.994 (0.986-1.001) |
| Charlson comorbidity index |  | 1.061 (0.998-1.028) |  | 1.096 (1.028-1.168) |
| Alcohol intake |  | 1.143 (0.791-1.649) |  | 1.444 (1.018-2.047) |
| Physical activity |  | 0.615 (0.399-0.948) |  | 0.717 (0.456-1.125) |

Multivariate adjusted model was adjusted for age, BMI, systolic BP, fasting blood glucose,total cholesterol, eGFR, Charlson comorbidity index, alcohol intake and physical activity.
