# Supplementary material for: The association of pancreatic cancer incidence with smoking status and smoking amount in Korean men
Source: Epidemiol Health. 2022 Apr 21;44:e2022040. doi: 10.4178/epih.e2022040 (PMC9350416; doi:10.4178/epih.e2022040)
Supplement: Supplementary Material 5. — Comparison between exclusion from analysis and inclusion in analysis [file epih-44-e2022040-suppl5.docx]

**Supplementary Material 5. Comparison between exclusion from analysis and inclusion in analysis**

| **Characteristic** | **Exclusion from analysis (N=4,230)** | **Inclusion in analysis (N=117,178)** | ***P*-value^*^** |
| --- | --- | --- | --- |
| Age (years) | 55.9 ± (8.2) | 57.5 ± (8.6) | <0.001 |
| BMI (kg/m^2^) | 24.0 ± (2.7) | 24.1 ± (2.7) | 0.175 |
| Systolic BP (mmHg) | 126.1 ± (14.3) | 126.4 ± (14.7) | 0.229 |
| Diastolic BP (mmHg) | 78.9 ± (9.7) | 78.8 ± (9.8) | 0.633 |
| Total cholesterol (mg/dL) | 196.1 ± (35.7) | 195.7 ± (36.5) | 0.406 |
| Triglyceride (mg/dL) | 158.3 ± (106.4) | 152.2 ± (103.0) | <0.001 |
| HDL-cholesterol (mg/dL) | 52.9 ± (30.4) | 53.2 ± (29.9) | 0.491 |
| LDL-cholesterol (mg/dL) | 115.4 ± (43.2) | 113.9 ± (38.5) | 0.028 |
| Fasting serum glucose (mg/dL) | 103.5 ± (25.9) | 103.1 ± (27.5) | 0.377 |
| SCr (mg/dL) | 1.44 ± (2.01) | 1.33 ± (1.75) | <0.001 |
| eGFR (mL/min per 1.73m^2^) | 80.9 ± (23.7) | 80.0 ± (20.9) | 0.013 |
| Alcohol intake (%) | 25.6 | 23.6 | 0.003 |
| Physical activity (%) | 18.0 | 18.3 | 0.647 |

Data are expressed as means (standard deviation) or percentages.

^*^*P*-value by t-test for continuous variables and Chi square test for categorical variables.
